# Unusual Presentation of Corynebacterium Endocarditis in a Patient Without Conventional Risk Factors: A Case Report

**DOI:** 10.7759/cureus.54970

**Published:** 2024-02-26

**Authors:** Abbas Mohammadi, Dima Youssef, Ashkan Mohammadi

**Affiliations:** 1 Internal Medicine, Valley Health System, Las Vegas, USA; 2 Medicine, University of Naples Federico II, Naples, ITA

**Keywords:** multidisciplinary management, mitral valve endocarditis, rare cardiac infections, pancytopenia, infective endocarditis, corynebacterium stratum

## Abstract

Infective endocarditis (IE) is a widespread condition marked by the infection of native or prosthetic heart valves, the endocardial surface, or an indwelling cardiac device. While native-valve IE is uncommon, patients with IE represent a diverse spectrum. Some respond well to treatment with few complications, while others face severe complications and an increased risk of mortality. Various factors contribute to this outcome, including delayed diagnosis, underlying health conditions like immunocompromised status or chronic diseases, and intravenous drug use. The most prevalent causes of IE are typically streptococci and staphylococci. IE attributed to *Corynebacterium *species is an exceptionally rare phenomenon, especially in individuals lacking conventional risk factors. This report presents a distinctive case involving *Corynebacterium *endocarditis in a 63-year-old female with a medical history encompassing intracranial aneurysm, hypothyroidism, and alcoholic cirrhosis. The patient's initial symptoms included shortness of breath, neck pain, and generalized weakness. Despite an initial focus on mild flu-like symptoms and a suspected urinary tract infection, subsequent evaluation unveiled pancytopenia and positive blood cultures for *Corynebacterium striatum*, culminating in the diagnosis of mitral valve endocarditis. This intricate clinical scenario, replete with numerous complications, underscores the significance of considering unusual pathogens in atypical presentations of IE. It prompts further exploration into the underlying mechanisms contributing to such infrequent occurrences.

## Introduction

Infective endocarditis (IE) is a severe and potentially life-threatening condition often associated with specific risk factors such as intravenous drug use, valvular heart disease, or prosthetic heart valves [1-3]. In recent studies conducted in the United States and Western Europe, the reported incidence of community-acquired native-valve endocarditis (NVE) ranges from 1.7 to 6.2 cases per 100,000 person-years with an upward trend worldwide [1,3,4]. Men are more frequently affected than women, with a mean male-to-female ratio of 1.7:1. Notably, among individuals with IE associated with injection-drug use, there is an observable trend toward younger age groups [1, 4]. The estimated incidence of IE in this specific population is approximately 150 to 2000 cases per 100,000 person-years, and it can be even higher among those individuals with known valvular heart disease [1].

Regarding complications, the rate of surgical treatment for IE is approximately 43-55%, while the in-hospital mortality rate ranges from approximately 20% to 26%. Other frequent complications include heart failure (38-56%), acute kidney injury (19-27%), and embolic events (22-41%) [2,3].

In the context of microbiology in IE, the most commonly identified microorganisms are *Staphylococcus* species and *Streptococcus* species, which collectively account for over 70% of the identified NVE cases, emerging as the most prevalent microorganisms [1]. Specifically, *Staphylococcus aureus* accounts for 31%, followed by viridans streptococci at 17%, enterococci at 11%, coagulase-negative staphylococci at 11%, *Streptococcus bovis* at 7%, and other streptococci (including nutritionally variant streptococci) at 5%. Non-HACEK (*Haemophilus*, *Aggregatibacter*, *Cardiobacterium*, *Eikenella*, *Kingella*) gram-negative bacteria, fungi, and HACEK each contribute 2%, with the remaining cases encompassing culture-negative endocarditis (8%), polymicrobial (1%), and various other organisms (3%) [2,3,5].

*Corynebacterium* species (known as diphtheroids) are aerobic and gram-positive bacilli typically regarded as non-pathogenic components of normal skin flora and mucosal membranes recognized as causing opportunistic disease in situations like in patients who are immunocompromised, have prosthetic devices, or have been in hospitals/nursing homes for long periods of time [6]. This infection commonly affects the left heart in adult males, with nearly one-third of patients having underlying valvular disease [7]. They have the capacity to induce life-threatening diseases in various clinical scenarios, including bacteremia and endocarditis [8,9]. Recent findings indicate that diphtheroids contribute to 4-9% of prosthetic valve endocarditis (PVE), but their involvement in NVE is comparatively rare, accounting for only 0.2-0.4% of cases [7].

In this report, we have a distinctive case of *Corynebacterium* NVE in an individual lacking traditional risk factors, adding an extra layer of uniqueness to the overall presentation. Literature on *Corynebacterium* endocarditis is limited, and the scarcity of reported cases warrants further investigation into the predisposing factors and pathogenic mechanisms associated with this specific presentation [7].

## Case presentation

A 63-year-old female residing in a long-term care facility, with a past medical history of intracranial aneurysm treated with coiling, cirrhosis, and hypothyroidism, presented to our hospital complaining of persistent cough, ongoing for a week, shortness of breath, neck pain lasting a few days, and generalized weakness. The neck pain started after a minor fall a few days prior. Due to worsening symptoms, including a worsening cough, she sought further evaluation at the hospital. Three months ago, she had a previous admission for spontaneous pulmonary hemorrhage originating from the left bronchus which was managed by bronchoscopy and endobronchial blockers. At that time, all tests and evaluations yielded unremarkable results, and she was discharged in good condition. Her medical records indicated a history of alcoholic cirrhosis. Physical examination was remarkable for a 3/6 systolic murmur best heard at left sternal border and decreased breath sounds in the base of both lungs. On arrival, she had a temperature of 36.5°C, a heart rate of 119/minute, blood pressure of 112/68 mmHg, respiratory rate of 17/minute, and a pulse rate of 97/minute.

**Figure 1:**
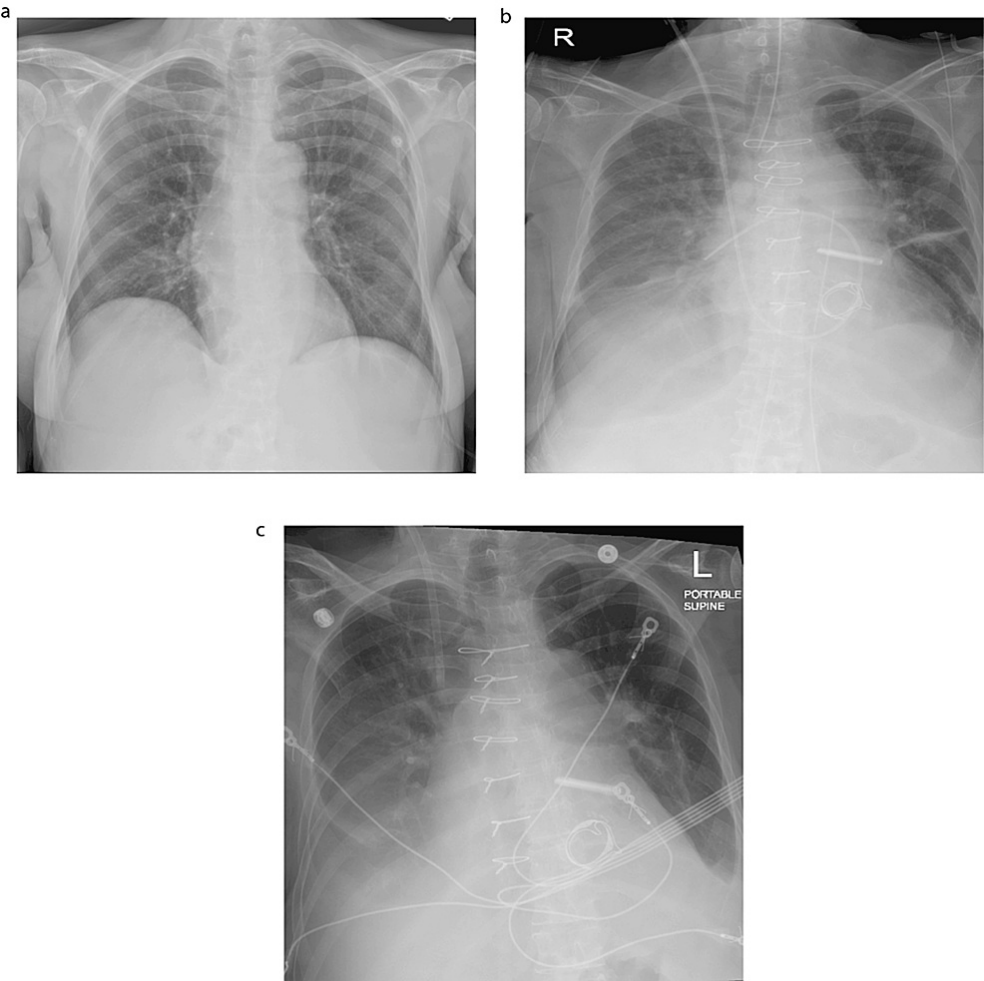
Sequential chest X-rays obtained at various points during the hospitalization (a) Day 1 CXR reveals no evidence of acute cardiopulmonary abnormalities; (b) On the 16th day post admission, following surgery, the CXR indicates a right-sided pleural effusion accompanied by right basilar atelectasis, along with subsegmental atelectasis in the left midlung field and left basilar atelectasis; (c) Day 20 CXR demonstrates stable cardiomegaly and infiltrates.

Initial labs revealed a white blood cell count (WBC) of 4.4x10^3^/mm³, hemoglobin of 12 g/dL, platelets of 81x10^3^/mcL, blood urea nitrogen (BUN) 15 mg/dL, creatinine 0.4 mg/dL, aspartate aminotransferase 14 units/L, alanine aminotransferase 9 units/L, and alkaline phosphatase 55 units/L. She tested positive for influenza B RNA. Urinalysis showed trace leukocyte esterase and 24 WBC suggested a urinary tract infection (UTI). Imaging studies, including CT scans of the cervical spine and head, as well as a chest X-ray, showed no acute changes (Figure 1a). Despite improvement in her cough, the patient still experienced neck pain. She was initially evaluated for flu-like symptoms, neck pain, UTI, and dehydration, leading to the initiation of ceftriaxone, oseltamivir, and IV fluids.

**Figure 2:**
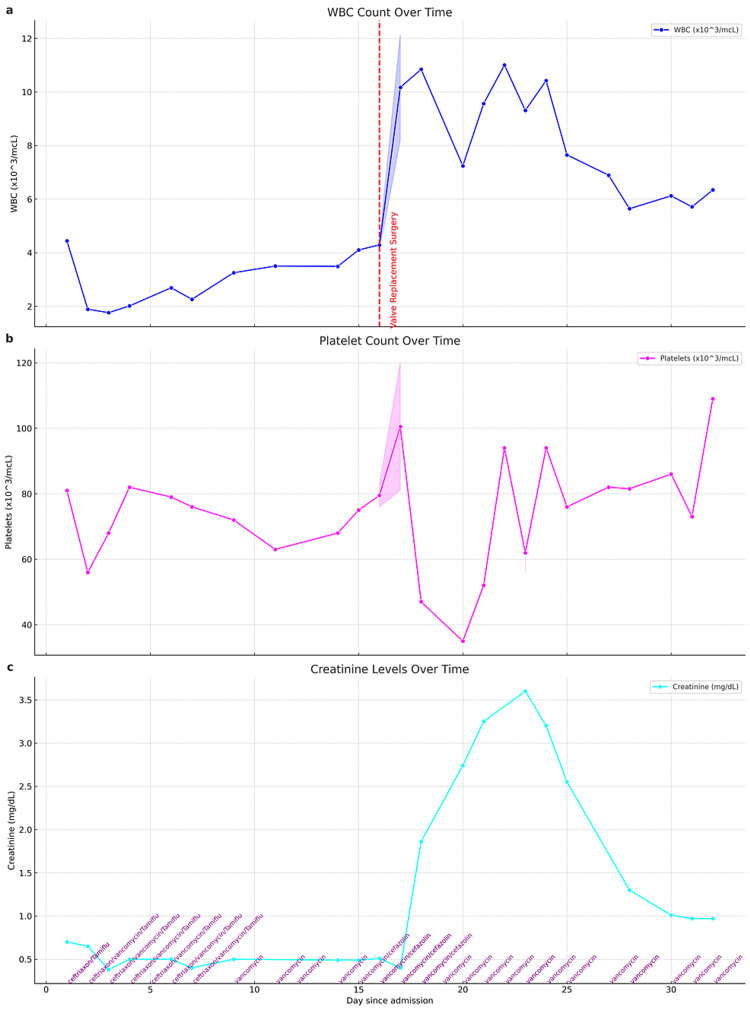
Three vertically aligned plots representing the WBC count, platelet count , and creatinine level over the course of hospital admission (a) WBC count (in x10^3^/mcL), marked by a blue line. Day 16 is highlighted with a red dashed vertical line, "Valve Replacement Surgery", indicating the day of the surgery; (b) Platelet count (in x10^3^/mcL) is depicted with a magenta line; (c) Creatinine levels (in mg/dL) is depicted with a cyan line. Text annotations in purple at the bottom of the plot indicate changes in the antibiotic regimen over the duration of the hospital stay, reflecting the adjustments in treatment. The x-axis represents "Day since admission" and the y-axis indicates the respective clinical parameter's units. The plot shows a comprehensive view of the patient's clinical progression, highlighting key treatment events and medication adjustments.

The next day, her WBC dropped to 1.9 (Figure 2a), hemoglobin to 9.5, and platelets to 56 (Figure 2b). She also had elevated procalcitonin (10.16 ng/ml) and C-reactive protein (7.8 mg/dl) prompted infectious disease consultation for her sepsis, resulting in the addition of vancomycin to ceftriaxone. Blood cultures isolated *Corynebacterium striatum* in all four blood samples drawn from the first day, with a repeat culture yielding *C. striatum* (blood drawn on day 4). The culture turned negative after three days of antibiotic treatment.

In response to acute persistent pancytopenia (Figures 2a, 2b), a hematologist was consulted, leading to comprehensive investigations, including flow cytometry, ultrasound of the abdomen, HIV testing, hepatitis panel, and anemia workup. Although daily improvement was observed in pancytopenia, it did not return to baseline values. Abnormal findings included an absolute reticulocyte count of 2.7 x10^4^/mcL, iron levels of 16 mcg/dL, total iron-binding capacity of 146 mcg/dL, transferrin saturation of 11%, and ferritin of 293 ng/mL. HIV and hepatitis tests were nonreactive, and flow cytometry revealed T cells within the normal range. Abdominal ultrasound demonstrated a heterogeneous liver and mild ascites, consistent with her history of cirrhosis. The hematologist recommended supportive transfusion of hemoglobin or platelet transfusion if needed and could not find any explanation for that. Continuous antibiotic treatment was advised to address the underlying source of sepsis.

**Figure 3:**
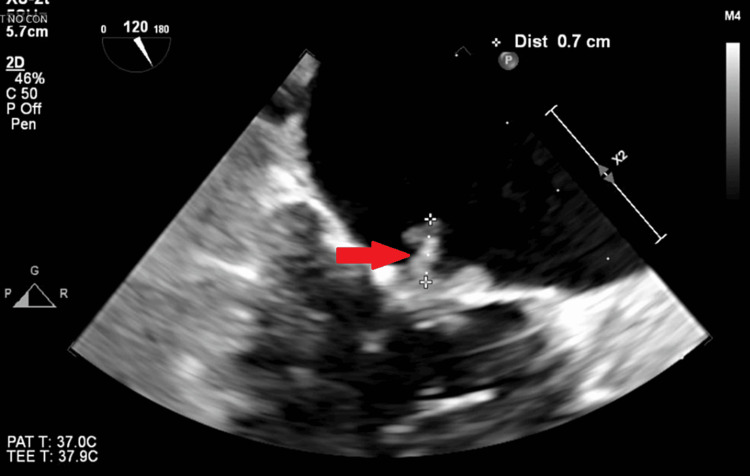
Transesophageal echocardiography six days after admission. Red arrow shows a valvular vegetation seen mainly on the anterior leaflet measuring 1 cm indicating mitral valve endocarditis

Despite significant improvement in symptoms and clear lungs observed on physical examination and chest X-ray, the patient still required 2-5 liters of oxygen through nasal cannula. Following positive blood culture results for *C. striatum*, transthoracic echocardiography was conducted the next day, revealing a normal ejection fraction, an enlarged left atrium, and severe mitral valve regurgitation with valvular vegetation measuring about 1 cm (Figure 3), indicative of mitral valve endocarditis. Transesophageal echocardiography confirmed severe regurgitation and revealed valvular vegetation. The cardiac surgeon was consulted, and their team initiated daily evaluations of the patient.

**Figure 4:**
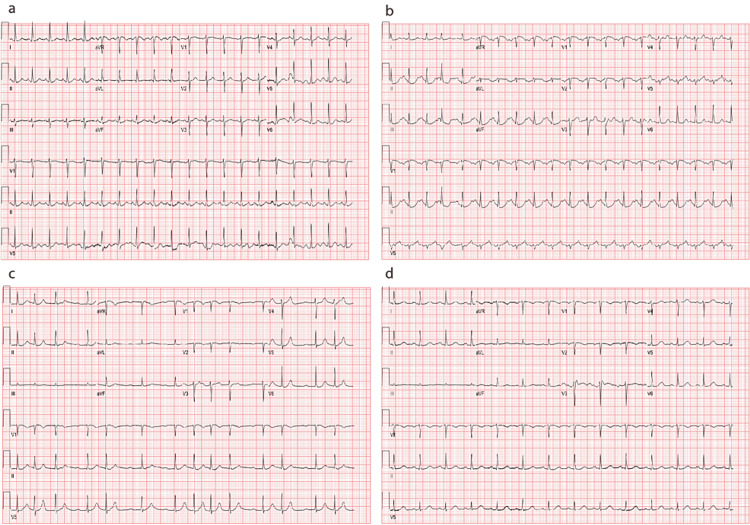
EKG records at different days after hospitalization (a) EKG from the day of admission showing sinus tachycardia; (b) EKG five days after admission shows prolonged PR and QT intervals; (c)Seven days after admission the EKG showed 1st degree AV block and episodes of paroxysmal atrial fibrillations shown on telemetry; (d) EKG was obtained from the last day, normal sinus rhythm.

A cardiologist consultation recommended mitral valve replacement. Coronary angiography was done and revealed mild angiographic coronary disease and no acute occlusion. Serial EKGs were performed, including baseline and discharge (Figure 4). On the fifth day of admission, the patient exhibited prolonged PR and QT intervals (Figure 4b). By the seventh day, the EKG revealed first-degree AV block, followed by episodes of paroxysmal atrial fibrillation (AF) detected on telemetry (Figure 4c). In response to the observed tachycardia episodes, the cardiologist initiated amiodarone treatment for the patient.

**Figure 5:**
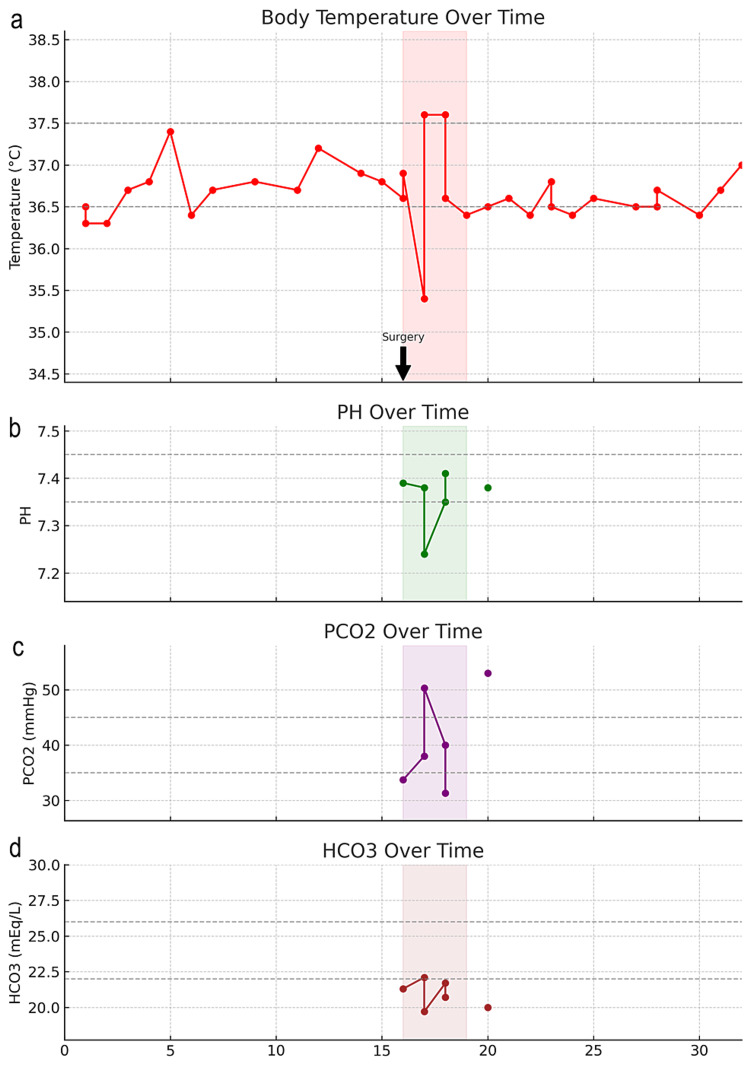
Clinical parameters over time from post-admission day one to day 20 (a) Daily body temperature of the patient, measured in °C. The normal body temperature range is indicated by the light gray dashed line. The graph indicates that the patient's temperature fluctuates and shows a significant drop below the normal range between days 16 and 19. This drop is highlighted in red and corresponds to the period during which the patient underwent surgery and subsequent ICU management. The black arrow labeled "Surgery" points to the beginning of this highlighted period; (b) pH level of the patient's blood. The normal pH range is indicated by a light gray dashed line. The green line represents the patient's pH levels. The green shaded area indicates a period of pH levels, which corresponds with the post-operative phase; (c) Partial pressure of carbon dioxide (PCO2) in the patient's blood over time, measured in mmHg. Normal PCO2 levels are indicated by the gray dashed line. The purple line indicates the patient's PCO2 levels, and the purple shaded area indicates status during the surgery and ICU management phase; (d) The bottom panel depicts the concentration of bicarbonate (HCO3) in the patient's blood over time, measured in mEq/L. Normal bicarbonate levels are shown by the gray dashed line. The brown line represents the patient's HCO3 levels, and highlighted in brown is during the surgery and ICU phase.

On the 16th day, following a comprehensive two-week workup, the cardiac surgeon performed an urgent mitral valve replacement, utilizing a 29 mm Edwards Mitris Resilia bovine pericardial prosthesis. In addition, a 35 mm Atriclip was used to ligate the left atrial appendage. Postoperatively, the patient's temperature decreased and she became acidotic (Figure 5a-5d); moreover, there was an unexpected rise in creatinine levels from 0.4 to 3.8 mg/dL (Figure 2). The hospitalist, in consultation with the pharmacist, adjusted the dosage of intravenous vancomycin to accommodate the acute kidney injury (AKI), planning for a treatment duration of six weeks from the date of surgery as recommended by the infectious disease consultant. A renal ultrasound indicated normal bilateral kidney size without any pathological findings. A nephrologist was consulted and diagnosed AKI, without any pre-existing chronic kidney disease. The AKI was presumed to be secondary to acute tubular necrosis (ATN) likely due to hypotension before or during the surgery.

After managing acute respiratory failure, which worsened post surgery, the patient transitioned from two liters of oxygen through nasal cannula to 50% fraction of inspired oxygen (FiO2) of high-flow oxygen for approximately five days, prompting ICU admission for acute infiltration (Figures 1b-1c). However, with supportive respiratory care, the patient improved and switched back to nasal flow cannula oxygen for the last two weeks of hospitalization. After managing hypotension, which commenced on the first day and fluctuated despite supportive care, with vasopressin and dopamine by the ICU team, kidney function improved and got back to baseline (Figure 2c). Eventually, she was successfully weaned off oxygen. A follow-up transthoracic echocardiogram two weeks post surgery revealed no remarkable findings. Her EKG converted to normal sinus rhythm by the time of discharge (Figure 3d), and she became totally asymptomatic, returning to her baseline and discharged to home.

## Discussion

This case challenges conventional perceptions of IE by depicting a rare manifestation involving *Corynebacterium* species in a patient lacking typical symptoms such as high fever, skin lesions, and night sweats, as well as conventional risk factors like heart valve abnormalities, prosthetic heart valves, previous instances of infective endocarditis, and intravenous drug use [5-7,9]. The prolonged course, atypical symptoms, and initial diverse diagnoses highlight the diagnostic complexities associated with uncommon pathogens. *Corynebacterium*, typically viewed as a skin commensal, can contribute to IE, emphasizing the need for a broader consideration of potential etiologies.

Bläckberg et al. conducted a comprehensive study on IE caused by *Corynebacterium* species, examining 30 cases. The most frequent pathogen was C. striatum, responsible for 37% of cases. Half of the patients required surgery, and the in-hospital mortality rate was 13%. Notably, 70% of cases involved prosthetic valve endocarditis. All *Corynebacterium* isolates were susceptible to vancomycin, while resistance to penicillin G was commonly observed [10]. Therefore, our patient presented a unique case of *Corynebacterium* NVE, which is notable due to the typically high mortality rate associated with this condition. However, through effective management, we successfully treated the patient, enabling her discharge to home. However, studies showed a 50% mortality rate for such cases, highlighting the success of our treatment approach [10,11].

This patient presented with a week-long cough and shortness of breath without remarkable fever and then tested positive for influenza test upon arrival at our hospital. This information, though suggestive of influenza and respiratory tract infection, could potentially mislead medical teams into neglecting further investigations, emphasizing the need to avoid anchoring on initial information in medicine, especially when typical IE risk factors are absent [12]. However, the presence of acute end-organ damage underscores the importance of conducting a comprehensive investigation rather than attributing everything solely to influenza or pneumonia. This highlights the significance of thorough examination and evaluation of patients, emphasizing a high suspicion for some potentially lethal conditions, especially considering that the majority of these patients may not exhibit typical symptoms or risk factors [1].

Importantly, the management of this patient was complicated by additional factors, adding a unique dimension to the case. Pancytopenia, potentially stemming from sepsis, viral pneumonia, or IE, was observed. The AKI could be attributed to the consequences from sepsis, or with low blood pressure after surgery. Additionally, the patient experienced AV block and new-onset AF. While the association between *Corynebacterium* IE and arrhythmias is not well-documented, theories suggest that infection of the valve and heart structures, and systemic inflammation, releasing high levels of cytokines, can disrupt electrical pathways [7,13,14]. Studies indicate that sepsis is linked to a six-fold higher risk of developing AF, also this patient tested positive for influenza, which aligns with research associating influenza infection with an 18% increased risk of AF [14,15]. Although pinpointing a single cause for these complications is challenging, the undeniable role of the severity of *Corynebacterium* IE in contributing to or triggering these issues is evident.

The complexity of this case was heightened by the patient's need for anticoagulation due to a prosthetic valve, juxtaposed with the contraindication arising from consistently low platelet counts in the range of 40-90x10^3^/mcL. Involving a nephrologist for AKI, consulting a pulmonologist and the ICU team for acute respiratory failure and hypotension, and identifying acute pancytopenia raise inquiries into the potential systemic effects of *Corynebacterium* IE. The patient's favorable response to antibiotic therapy, coupled with the subsequent improvement in pancytopenia and renal function, implies a correlation between the infective process and these systemic abnormalities.

Following the evaluation by a vascular surgeon for severe mitral regurgitation, the patient met the criteria for urgent valve replacement. Post surgery and after multidisciplinary team effort, a swift return to her baseline occurred within a few days. This case report suggests managing such a life-threatening infection necessitates a multidisciplinary approach involving healthcare providers from diverse backgrounds.

Since *Corynebacterium* species are often mistaken for contaminants, distinguishing them as a cause of IE can be challenging. It is advisable to conduct echocardiography to assess for valvular issues if it is detected in blood stream, thereby helping to confirm or rule out IE in cases where *Corynebacterium* is detected. This approach is crucial for accurate diagnosis and management. The increasing antibiotic resistance of corynebacteria poses a significant challenge to treatment, making this a concerning issue in healthcare [10,16].

Additional investigations are necessary to uncover the predisposing factors and underlying mechanisms that contribute to *Corynebacterium* IE. It is crucial to bear in mind that bacteremia caused by gram-positive rods can have severe consequences. Physicians should prioritize IE in their differential diagnoses when encountering such cases and take prompt and decisive actions.

## Conclusions

This study presents a rare case of *Corynebacterium* endocarditis devoid of conventional risk factors and manifesting atypical features. This highlights the necessity for increased awareness and research into its mechanisms, particularly in non-typical presentations, to enhance diagnostic and therapeutic strategies. Testing for IE is vital in patients with sepsis and *Corynebacterium* bacteremia, emphasizing the importance of avoiding the label of culture contamination, especially when the antibiotic response is suboptimal.
